# Implementing a Personalized Music Intervention for Persons Living With Dementia in Nursing Facilities

**DOI:** 10.1093/geroni/igab046.902

**Published:** 2021-12-17

**Authors:** Megumi Inoue, Meng-Hao Li, Emily Ihara, Catherine Tompkins, Christi Clark, Shannon Layman

**Affiliations:** 1 George Mason University, Fairfax, Virginia, United States; 2 George Mason University, Arlington, Virginia, United States; 3 Harpeth Consultant Advisory Group, Franklin, Tennessee, United States

## Abstract

The Mason Music & Memory Initiative (M3I) team has implemented a personalized music intervention in nursing facilities across Virginia aiming to improve behavioral and psychological symptoms of persons living with dementia. This person-centered intervention uses a unique music playlist comprising songs, artists, and preferred musical genres. The preliminary findings from a randomized controlled trial will be reported, the purpose of which was to examine the intervention impact on nursing home residents’ mood and behavior. Based on the findings from 16 facilities with 158 residents who have completed the study, both quantitative and qualitative data indicate the positive effects on residents, including improved sleep and mood, as well as reduced agitation. The challenges in implementing intervention research in nursing facilities during the COVID-19 pandemic and the principles of telehealth and virtual support for facilities that were used to address those challenges will also be discussed.

